# Thyroid fine-needle aspiration trends before, during, and after the lockdown: what we have learned so far from the COVID-19 pandemic

**DOI:** 10.1007/s12020-020-02559-z

**Published:** 2020-12-07

**Authors:** Raffaele Palladino, Ilaria Migliatico, Roberta Sgariglia, Mariantonia Nacchio, Antonino Iaccarino, Umberto Malapelle, Elena Vigliar, Domenico Salvatore, Giancarlo Troncone, Claudio Bellevicine

**Affiliations:** Department of Public Health, University of Naples Federico II, Via S. Pansini 5, 80131 Naples, Italy

**Keywords:** Thyroid, Fine-needle aspiration, COVID-19, Patient prioritisation

## Abstract

**Purpose:**

Nowadays, the clinical management of thyroid nodules needs to be multi-disciplinary. In particular, the crosstalk between endocrinologists and cytopathologists is key. When FNAs are properly requested by endocrinologists for nodules characterised by relevant clinical and ultrasound features, cytopathologists play a pivotal role in the diagnostic work-up. Conversely, improper FNA requests can lead to questionable diagnostic efficiency. Recently, recommendations to delay all non-urgent diagnostic procedures, such as thyroid FNAs, to contain the spread of COVID-19 infection, have made the interplay between endocrinologists and cytopathologists even more essential. The objective of this study was to assess the impact of COVID-19 pandemic on our practice by evaluating the total number of FNAs performed and the distribution of the Bethesda Categories before, during, and after the lockdown.

**Methods:**

We analysed the FNA trends before (1st January 2019 to March 13th 2020), during (March 14th to May 15th), and after (May 16th to July 7th) the lockdown.

**Results:**

Although the total number of weekly FNAs dropped from 62.1 to 23.1, our referring endocrinologists managed to prioritise patients with high-risk nodules. In fact, in the post-lockdown, the weekly proportion of benign diagnoses dropped on average by 12% and that of high-risk diagnoses increased by 6%.

**Conclusions:**

The lesson we have learned so far from this pandemic is that by applying safety protocols to avoid contagion and by increasing the threshold for FNA requests for thyroid nodules, we can continue to guarantee our services to high-risk patients even in times of a health crisis.

## Introduction

The recent COVID-19 outbreak has had great consequences on our healthcare system. At the beginning of the outbreak, Italy was the second most impacted country after China—a phenomenon that led to a national lockdown [1]. Such drastic containment measure led to the postponement of non-urgent and elective medical and surgical procedures, among these FNA cytology for thyroid nodules [2, 3]. It is now well established that clinical management of thyroid nodules needs to be multi-disciplinary. In particular, a direct crosstalk between endocrinologists and cytopathologists is key [4]. When thyroid fine-needle aspiration is properly requested by endocrinologists for nodules with high-risk ultrasound (US) features, this diagnostic technique can be highly instrumental in the diagnostic work-up of thyroid nodules, especially when performed by a trained cytopathologist [5]. Conversely, when it is improperly requested, thyroid FNA can be detrimental, for it can lead to unnecessary diagnostic thyroid surgeries and to lower diagnostic efficiency [6, 7]. Indeed, in an attempt to limit the number of unnecessary requests for FNAs, particularly for nodules with low-risk US features, the recent guidelines for the management of thyroid nodules have modified the criteria for FNA recommendation [8, 9]. However, these general recommendations are not always met in clinical routine practice. Indeed, several surveys on the actual practice patterns of American and European endocrinologists have shown that the criteria for FNA requests are less stringent than those generally recommended [10, 11].

During the current health crisis, unnecessary requests for thyroid FNA are even less justifiable and should be avoided for two main reasons. First, as we just mentioned, the majority of FNA procedures can be safely postponed, and second, the handling of aspirated material can expose professional staff to potential COVID‐19 transmission [2, 3, 12]. In fact, in such critical scenario, thyroid FNA can no longer be considered ʻroutine’ because cytopathologists must work in tandem to restrict to a minimum their contact time with patients and to ensure safe handling of potentially infectious fresh specimen [13, 14].

Located at Federico II University of Naples, our US-guided FNA clinic is run by experienced interventional cytopathologists and represents a privileged vantage point from which to evaluate the most recent thyroid FNA trends—the reason being that patients are referred to us from various intramural endocrinology departments and nearby community hospitals [5].

The aim of this study was to assess how the COVID-19 pandemic has changed our practice. To this aim, we evaluated the total number of FNAs performed and the distribution of the Bethesda System for Reporting Thyroid Cytopathology diagnostic categories (TBSRTC DCs) before, during, and after the lockdown. In particular, we reviewed the distribution of the TBSRTC DCs from January 2019 to July 2020 to assess whether during this time period the clinical guidelines recommended to limit FNA requests were actually met in our routine clinical practice to prioritise high-risk patients.

## Materials and methods

The laboratory information database system was searched to obtain the total number of thyroid FNAs performed, along with the diagnostic reports rendered from January 2019 to July 2020. Overall, 3684 US-guided thyroid FNA samples were retrieved. The patients’ mean age was 52.9 (SD 14.2); the majority were females (78.3%). The US-guided thyroid FNAs were mostly performed by two interventional cytopathologists (C.B., E.V.), as described in our previous experience [5]. Generally, before the pandemic, we would perform one FNA pass for each nodule, adopting, when feasible, the Zajdela technique (without suction) to avoid excessive blood contamination [5, 15]. The smears would then be air-dried and stained with Diff-Quik (Bio Optica S.p.A, Milan, Italy). However, when the pandemic took hold in our country, we abandoned the air-drying method of ethanol fixation, since air-dried slides could generate aerosols and droplets containing viable and transmissible viruses. Moreover, rapid on-site evaluation (ROSE) was performed only when strictly necessary, preferring immediate methanol fixation rather than air-drying for Diff-Quik staining [14]. However, when air-drying was deemed necessary, the smears were managed in a biosafety cabinet to avoid aerosolisation. All cytopathologists and lab technicians wore personal protective masks with a respirator filter and face shields to protect their eyes [16]. All thyroid FNAs were classified adopting the TBSRTC terminology [17]. This study was approved by the Ethics Committee ʻCarlo Romano,’ of the University of Naples Federico II (protocol 155/15/ES1). Written consent was obtained from each patient after full explanation of the purpose and nature of all procedures.

### Statistical analysis

The study period was divided into three phases: (1) the pre-lockdown phase, from 1st January 2019 to March 13th 2020; (2) the lockdown phase (i.e. phase I), from March 14th 2020 to May 15th 2020; (3) the post-lockdown phase (i.e. phase 2), from May 16th to July 7th. Differences in the number of FNAs performed at Federico II University Hospital (Naples, Italy) before and after the lockdown were assessed with an interrupted time series analysis without external control [18, 19]. Differences in the number of FNAs performed per week were modelled using the Poisson regression model. Although the model was adjusted to include the lockdown period as step change, it was not adjusted for additional variables such as age, sex, and area of residence. Indeed, adding these variables would not have improved the goodness of fit of the model, which was tested with the Akaike Information Criterion and the Bayesian Information Criterion. A preliminary data analysis suggested that no adjustment was required for autocorrelation of the error term. The model did not consider lags because the implementation of the lockdown had an immediate effect on the number of FNAs performed as outpatient visits, which were indeed suspended except for urgent cases [13]. Throughout the study period, all FNAs performed were analysed and stratified according to the TBSRTC DCs.

To determine the trends of FNA requests for low-risk and high-risk clinical and ultrasonographic thyroid nodules, we evaluated the distribution of benign, suspicious for malignancy (SFM), and malignant FNAs diagnosed during the study period. In fact, these TBSRTC DCs show sufficient low (benign) and high (SFM and malignant) risk of malignancy (ROM) and can be adopted as indicators [9, 20].

Conversely, TBSRTC DCs atypia of undetermined significance/follicular lesion of undetermined significance (AUS/FLUS) and follicular lesion/suspicious for follicular lesion (FN/SFN), which are associated with a low to intermediate ROM [21], were both considered as indeterminate diagnostic categories. Analyses were presented as incidence rate ratios (IRRs) and 95% confidence intervals (CIs). Moreover, we evaluated the average number of total FNAs performed per week and the TBSRTC DCs diagnosed throughout the study period.

## Results

**Fig. 1:**
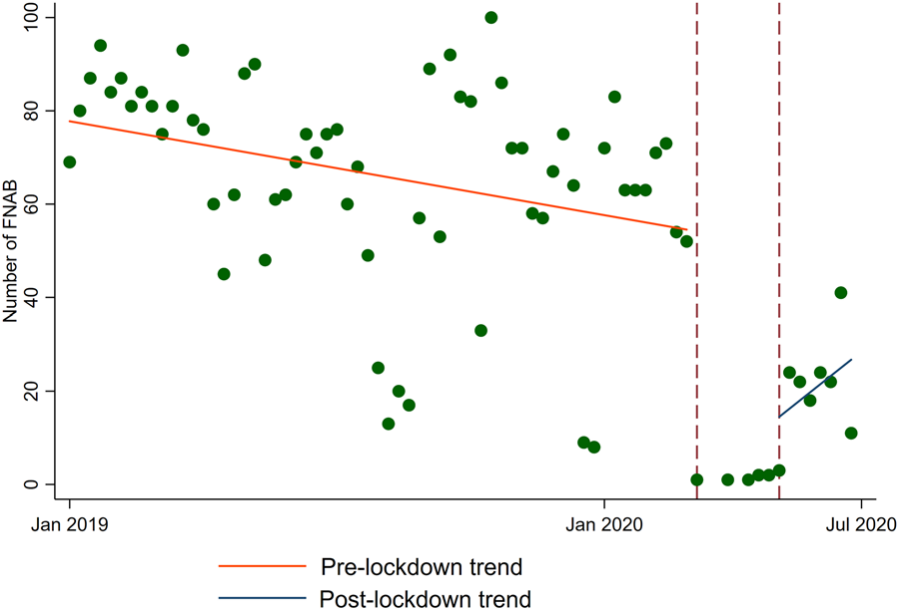
Trends of total FNA performed before, during and after the national lockdown

**Fig. 2:**
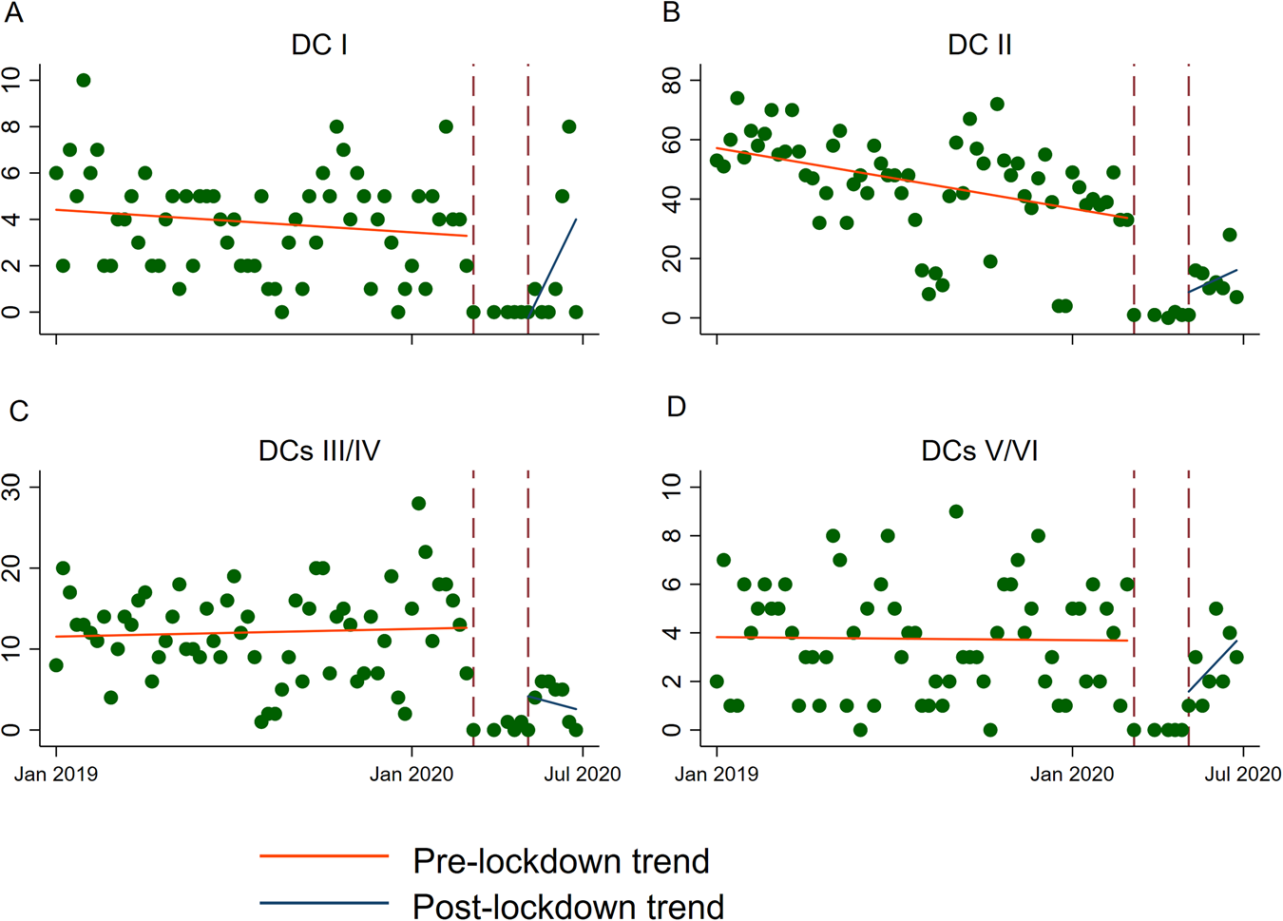
Bethesda diagnostic categories (DCs) trends before, during and after the lockdown. **A** Inadeguate/unsatisfactory (DC I), **B** benign (DC II), **C** indeterminate (DCs III/IV), **D** suspicious for malignancy and malignant (DCs V/VI)

Both the average number and the IRRs of total thyroid FNAs and TBSRTC DCs diagnosed per week are summarised in Table 1. Total FNA and TBSRTC DC trends before, during, and after the lockdown are shown in Figs 1, 2.

**Table 1:** Average weekly number (AWN) and trends expressed as incidence rate ratio (IRR) of benign, suspicious and malignant (SFM + MAL), indeterminate (AUS/FLUS and FN/SFN), inadequate/unsatisfactory cytological diagnoses and total thyroid FNA

	Pre-lockdown			Lockdown			Post-lockdown		
	AWN (SD)	IRR (95% CI)	*p* value	AWN (SD)	IRR (95% CI)	*p* value	AWN (SD)	IRR (95% CI)	*p* value
Benign	44.7 (17.0)	0.99 (0.99–1.00)^a^	<0.001	1 (0.7)	0.12 (0.08–0.17)^a^	<0.001	14 (6.9)	1.28 (1.19–1.37)^a^	<0.001
SFM + MAL	3.7 (2.3)	0.99 (0.99–1.00)	0.896	0.2 (0.4)	0.18 (0.08–0.42)^a^	<0.001	2.9 (1.3)	1.32 (1.12–1.57)^a^	<0.001
Indeterminate	11.9 (5.7)	1.00 (0.99–1.00)	0.447	0.4 (0.5)	0.13 (0.07–0.23)^a^	<0.001	3.9 (2.4)	1.12 (0.98–1.29)	0.102
Inadequate/unsatisfactory	3.8 (2.2)	0.99 (0.99–1.00)	0.194	0 (0)	0.05 (0.01–0.23)^a^	<0.001	2.1 (3.1)	1.62 (1.28–2.06)^a^	<0.001
Total thyroid FNA	62.1 (23.0)	0.99 (0.99–1.00)^a^	<0.001	1.8 (0.8)	0.12 (0.09–0.16)^a^	<0.001	23.1 (9.1)	1.27 (1.21–1.35)^a^	<0.001

*SD* standard deviation, *CI* confidence interval

^a^Statistically significant results

Before the lockdown, from January 2019 to March 13th 2020, a total of 3493 thyroid FNAs were performed, with an average of 62.1 FNAs per week (Table 1). During this period, the trend of FNAs performed in our clinic was slightly reduced by ~1% each week (IRR 0.99, 0.99–1.00, Fig. 1). This slight but statistically significant reduction was also observed for benign cytological diagnoses (IRR 0.99, 0.99–1.00, Fig. 2B) but not for inadequate, indeterminate and high ROM TBSRTC DCs (Table 1, Fig. 2A, C, D).

During the lockdown period (from March 14th to May 15th), the number of FNAs performed in our clinic drastically dropped to 19 FNAs, with an average of 1.8 weekly FNA (Table 1). Consistently, such decreasing trend affected all TBSRTC DCs (Fig. 2).

Our FNA clinic fully reopened at the beginning of the post-lockdown. A total of 182 thyroid FNAs were performed from May 16th to July 7th, featuring, on average, 23.1 weekly FNAs (Table 1). As expected, positive trends were observed for both the total number of FNAs performed (IRR 1.27, 1.21–1.35, Fig. 1) and the TBSRTC DCs (Fig. 2A, B, D), except for the indeterminate (AUS/FLUS and FN/SFN) DCs (Fig. 2C). The incidence rate ratio between benign and high ROM DCs (SFM and Malignant) changed between the pre-lockdown and post-lockdown periods. During the pre-lockdown period, the average benign diagnoses per week were 44.7 out of 62.1 total FNAs, representing 72% of FNA diagnoses. Conversely, the SFM and Malignant DCs were 3.7, representing 6% of FNA diagnoses. Instead, during the post-lockdown period, the number of benign FNA diagnosed per week was 14 out of 23.1 total FNAs, representing 60% of all FNA diagnoses; by contrast, the number of high ROM DCs was 2.9, representing 12% of all FNA diagnoses (Table 1).

## Discussion

When a thyroid nodule is diagnosed, FNA plays a key role in ruling out the presence of cancer. An FNA clinic run by dedicated interventional cytopathologists, who personally perform US-guided FNAs, is a cost-effective and time-saving solution to lower the incidence of inadequate/unsatisfactory samples [5]. However, the diagnostic accuracy of FNA depends not only on the correct sampling procedure and specimen handling, but also on proper FNA requests. In fact, performing FNA indiscriminately in a population with a high prevalence of thyroid nodules may lead to a low cost-benefit ratio, which, in turn, may result in lower diagnostic efficiency and high frequency of inadequate/unsatisfactory FNA [7, 22].

The overuse of US screening in asymptomatic patients coupled with a low threshold for thyroid FNA requests can create a time and economic burden not always sustainable by an FNA clinic. Although interventional cytopathologists performing US-guided by themselves are trained to interpret the US, along with the microscopic features of the sampled thyroid nodules [23], hardly ever do they interfere with the clinicians’ request for an FNA even when they recognise improper patient selection. Thus, multi-disciplinary efforts between endocrinologists and cytopathologists are necessary to improve clinical decision making [4].

This is even more true in the midst of the current pandemic. Not surprisingly, both the European clinical guidance documents on the management of endocrine conditions in the time of COVID-19 and the U.S. Endocrinology Division of Phoenix Veteran Affairs Healthcare System recommendations, clearly state that most thyroid FNAs can be safely postponed [2, 3].

Whereas many healthcare services were totally disrupted during the lockdown, our Department continued to provide diagnostic support to high-risk patients. Thanks to a telematic support system, which allowed us to analyse patients’ relevant medical documentation, and to online video consultations with referring clinicians, we were able to take a proactive role in prioritising patients who could not be deferred [13]. Consequently, during the lockdown phase, requests for FNA were relatively higher in patients at high oncological risk, including patients with breast lumps or pathological lymph node enlargements, than in patients with thyroid nodules [16].

Globally, we observed a drastic drop in the number of thyroid FNA requests (Table 1, Figs 1, 2), as evidenced by the fact that only 19 FNAs were performed, with a weekly average of 1.8 thyroid aspirates. This is in line with recent international experiences in which a significant drop in thyroid FNA during the COVID-19 pandemic was observed [24, 25].

In the post-lockdown phase (phase 2), stringent procedural protocols, recommended by our regional healthcare system, were applied in our FNA clinic. Face to face consultations were cancelled. Indeed, all patients requiring thyroid FNA were triaged by telephone before FNA procedure. Calls were also made to ask whether patients had experienced or were experiencing any COVID-19-related symptoms. Temperature checks were done on every patient entering our waiting room, which was equipped with hydroalcoholic solution for hand disinfection. Moreover, rapid serology assays were also performed and patients with positive/doubtful results were referred to the Infectious Disease Department for further analysis. Facial masks were required throughout the entire procedure; all family members were asked to wait in the waiting room.

The implementation of these restrictive protocols more than halved the number of FNAs routinely performed at our institution. In fact, during phase 2, FNAs dropped from 62.1 to 23.1 weekly (Table 1). Interestingly, the proportion between benign and high ROM TBSRTC DCs was also different during these two phases. In fact, whereas in the post-lockdown, the proportion of benign FNAs on the total decreased, on average, by 12%, that of SFM and Malignant (high ROM DCs) FNAs increased by 6%. Although we acknowledge that we evaluated only the first 8-week period of the post-lockdown phase, these figures are nonetheless indicative of the efforts made by referring endocrinologists to avoid unnecessary FNAs and, thus, to comply with the recent guidelines on the management of thyroid nodules. Indeed, we argue that the higher number of SFM and Malignant FNAs performed during the post-lockdown phase reflects the higher number of high-risk nodules referred to our FNA clinic.

Another intriguing observation arising from this study was that the slight drop in the total number of FNAs performed before the lockdown, (Fig. 1) was paralleled by a similar reduction in the number of benign FNAs (Fig. 2B). These trends indicate that shortly before the emergency, our referring endocrinologists had already begun to apply higher thresholds for FNA requests—a trend that was further encouraged by the need to restrict the number of outpatients visits to prevent the risk of COVID-19 transmission.

Interestingly, although the drop in FNAs observed during the lockdown phase was similar for all TBSRTC DCs, the FNA trend for indeterminate diagnoses remained the same during the pre- and post-lockdown phases (Table 1, Fig. 2C). This observation can be explained by the fact that diagnosing low to intermediate risk DCs, namely AUS/FLUS and FN/SFN, is highly challenging owing to the poor reproducibility of their microscopic criteria [26]. Moreover, the recent reclassification of encapsulated follicular variant of papillary thyroid carcinoma as NIFT-P, which is often classified as indeterminate, increases the diagnostic challenges of these TBSRTC DCs [27]. Since our institution routinely submits indeterminate cases to an intra-institutional review strategy, whose purpose is to limit inter-observer variations [28], the trends of these DCs remained stable in the pre- and post-lockdown periods.

Finally, we noticed that the trend of inadequate/unsatisfactory samples was low and stable in the pre-lockdown phase (Fig. 2A), reinforcing the notion that interventional cytopathologists, besides having the skills for US guidance, are also able to ensure specimen adequacy thanks to their expertise in smearing techniques and ROSE of aspirated material [5]. However, even though we observed a significant positive trend in the number of inadequate/unsatisfactory FNA during the phase 2 (Fig. 2A), the weekly average number of inadequate samples was similar to that observed between the pre-lockdown (3.8 out of 62.1, 6.1%) and the post-lockdown periods (2.1 out of 23.1 total FNA, 9%).

We cannot ignore the fact that the FNA trends seen in our study might have been influenced by some psychological factors, mainly fear of the virus. Indeed, such fear made many patients, especially the elderly, reluctant to see their endocrinologists or to undergo any type of medical procedure. In fact, in the subset of older patients (>65 years old), we did not observe a significant positive trend of total FNA performed during the post-lockdown period (IRR 0.92, 0.80–1.06). Such phenomenon highlights the complex mechanism behind patient selections for FNA procedures in the time of a pandemic. Indeed, we have learned that when clinicians select patients for FNA procedures during a crisis like the current pandemic, they should factor in mechanisms that include not only the clinical and US features of thyroid nodules but also patients’ anxiety and fear of getting infected.

## Conclusions

The COVID-19 pandemic has changed many aspects of our personal and professional lives. Despite the short duration of our study period, we demonstrated that our referring endocrinologists managed to prioritise the procedures for patients with high-risk thyroid nodules. Concomitantly, despite implementing new protocols to avoid the spread of COVID-19 infection, interventional cytopathologists were able to perform and interpret thyroid FNAs thanks to their valuable expertise.
